# 26S Proteasome Non-ATPase Regulatory Subunits 1 (PSMD1) and 3 (PSMD3) as Putative Targets for Cancer Prognosis and Therapy

**DOI:** 10.3390/cells10092390

**Published:** 2021-09-11

**Authors:** Andres J. Rubio, Alfonso E. Bencomo-Alvarez, James E. Young, Vanessa V. Velazquez, Joshua J. Lara, Mayra A. Gonzalez, Anna M. Eiring

**Affiliations:** 1 Paul L. Foster School of Medicine, Texas Tech University Health Sciences Center at El Paso, El Paso, TX 79905, USA; Andres.J.Rubio@ttuhsc.edu (A.J.R.); vanesvel@ttuhsc.edu (V.V.V.); Joshua.J.Lara@ttuhsc.edu (J.J.L.); 2 Center of Emphasis in Cancer, Department of Molecular and Translational Medicine, Paul L. Foster School of Medicine, Texas Tech University Health Sciences Center at El Paso, El Paso, TX 79905, USA; alfonso.e.bencomo@ttuhsc.edu (A.E.B.-A.); mayra.a.gonzalez@ttuhsc.edu (M.A.G.); 3 Graduate School of Biomedical Sciences, Texas Tech University Health Sciences Center at El Paso, El Paso, TX 79905, USA; James01.Young@ttuhsc.edu

**Keywords:** oncogenes, drug targets, proteasome inhibition, cancer

## Abstract

Ever since the ubiquitin proteasome system was characterized, efforts have been made to manipulate its function to abrogate the progression of cancer. As a result, the anti-cancer drugs bortezomib, carfilzomib, and ixazomib targeting the 26S proteasome were developed to treat multiple myeloma, mantle cell lymphoma, and diffuse large B-cell lymphoma, among others. Despite success, adverse side effects and drug resistance are prominent, raising the need for alternative therapeutic options. We recently demonstrated that knockdown of the 19S regulatory components, 26S proteasome non-ATPase subunits 1 (*PSMD1*) and 3 (*PSMD3*), resulted in increased apoptosis of chronic myeloid leukemia (CML) cells, but had no effect on normal controls, suggesting they may be good targets for therapy. Therefore, we hypothesized that *PSMD1* and *PSMD3* are potential targets for anti-cancer therapeutics and that their relevance stretches beyond CML to other types of cancers. In the present study, we analyzed *PSMD1* and *PSMD3* mRNA and protein expression in cancerous tissue versus normal controls using data from The Cancer Genome Atlas (TCGA) and the Clinical Proteomic Tumor Analysis Consortium (CPTAC), comparing expression with overall survival. Altogether, our data suggest that PSMD1 and PSMD3 may be novel putative targets for cancer prognosis and therapy that are worthy of future investigation.

## 1. Introduction

The ubiquitin proteasome system (UPS) is a highly regulated, multi-enzyme complex that allows cells and tissues to maintain protein homeostasis [1]. It is comprised of a series of enzymes that post-translationally modify proteins with an ubiquitin chain, tagging them for destruction via the 26S proteasome [2]. The 26S proteasome is composed of two main subcomplexes, the 19S regulatory complex and the 20S core complex. The proteasome regulates cellular protein levels through the binding of ubiquitylated proteins to the 19S regulatory complex, followed by catalytic degradation in the 20S core complex. Depending on the demands of the cell, the protein turnover rate fluctuates in part through activity of this biological system [2,3].

In diseases such as cancer, the UPS can go rampant due to the extreme requirements of malignant cells. As cancerous cells rapidly divide, proteins are made and destroyed at an exuberant rate, leading to deteriorating effects such as cachexia and fatigue [4]. There have been several successful attempts at producing cancer treatments directly targeting the 26S proteasome, the first example being bortezomib (BTZ), which was first approved by the FDA in 2003 to treat multiple myeloma (MM) [5]. BTZ has found its success as a reversible inhibitor by binding to the β5 subunit of the 20S core complex to inhibit its chymotrypsin-like enzymatic activity. This inhibition causes an accumulation of ubiquitylated proteins and subsequent caspase-mediated apoptosis of myeloma cells [6]. Since its approval, the use of BTZ has been widened to treat other forms of cancers, such as relapsed mantle cell lymphoma, diffuse large B cell lymphoma, colorectal carcinoma, and thyroid carcinoma, which has effectively demonstrated that the UPS can be a suitable target for cancer therapy [7,8,9,10]. Even when treatment is successful, however, a narrow therapeutic window exists along with a high degree of toxicity [11]. Individuals with relapsed or resistant multiple myeloma experience peripheral neuropathy after taking BTZ, which greatly affects the quality of life for patients [12]. Other obstacles faced by currently FDA approved proteasome inhibitors are poor bioavailability in solid tumors, solubility, developed or acquired resistance, or adverse side effects such as anemia, hepatic toxicity, and adverse cardiovascular events [11,13]. BTZ, like other anti-cancer therapies, is subject to the unfortunate outcome of drug resistance [14]. Acquired tolerance to BTZ in cancer has led to the development of second-generation proteasome inhibitors, such as carfilzomib and ixazomib, which are more selective and specific [15,16]. Despite the success demonstrated by these treatments, adverse side effects and drug resistance are still prominent, raising the need for alternative therapeutic options. One option is the use of combination therapies aimed at reducing the concentration of BTZ. This was recently demonstrated in hepatocellular carcinoma and mammary adenocarcinoma cells with a combination of BTZ and menadione sodium bisulfate (MSB, Vikasolum, vitamin K3). Astakhova et al. demonstrated that combination therapy with MSB in addition to BTZ required BTZ doses that were four to ten times lower than its clinical dose [17]. Despite this success, there is a great need for inhibitors that can target solid tumors, have better bioavailability, and are less toxic compared with currently available treatment options.

Since the UPS is composed of a series of highly regulated proteins, there is ample opportunity to discover new targets for cancer therapy. Indeed, the expression of many different proteasome subunits are reported to be altered in multiple different cancers, including human papillary thyroid carcinoma [18], breast cancer [19,20,21,22,23], colon cancer [24,25], and ovarian cancer [26], among others [27,28,29,30]. We recently demonstrated that two members of the 19S regulatory complex, 26S proteasome non-ATPase subunits 1 (PSMD1) and 3 (PSMD3), may be potential targets for therapy in tyrosine kinase inhibitor (TKI)-resistant chronic myeloid leukemia (CML) cells. In CML, *PSMD1* and *PSMD3* mRNA expression was notably increased in patients that had transitioned from the chronic phase to the blast phase of the disease. Knockdown of either protein resulted in reduced survival of CML cell lines and patient samples without affecting CD34^+^ cord blood controls, suggesting they may be good targets for therapy [31]. In breast cancer, PSMD1 was shown to regulate cell growth by inducing p53 protein degradation, thereby promoting cell cycle progression [22]. Similarly, PSMD3 was shown to be overexpressed at the protein level in human epidermal growth factor receptor 2 (HER2)-positive breast cancer, helping to stabilize HER2 and culminating in a worse overall survival (OS). Knockdown of PSMD3 led to reduced cell proliferation, reduced colony formation, and increased apoptosis in breast cancer cell lines [23], and co-silencing of PSMD3 and HER2 resulted in additive inhibition of cell viability and promoted apoptosis in breast cancer [32].

Based on the variability of RNA and protein expression in cancer, the inadequacy of many proteasome inhibitors, and our recent observations in CML, we hypothesized that *PSMD1* and *PSMD3* are potential targets for anti-cancer therapeutics and that their relevance stretches beyond CML to other types of cancers. In the present study, we comprehensively analyzed the expression and prognostic value of *PSMD1* and *PSMD3* across multiple cancer types using data from The Cancer Genome Atlas (TCGA) [33] and the Clinical Proteomic Tumor Analysis Consortium (CPTAC) [34,35], comparing expression with OS. Our analysis revealed that differential expression of *PSMD1* and *PSMD3* is correlated with worse OS in several different cancer types. Ultimately, we highlight PSMD1 and PSMD3 as potential therapeutic targets for the development of novel proteasome inhibitors to treat cancer patients with less toxicity. Additionally, *PSMD1* and *PSMD3* may be novel prognostic biomarkers for cancers affecting multiple different tissue types.

## 2. Materials and Methods

### 2.1. Analysis of PSMD1 and PSMD3 Differential Expression across TCGA Cancers Compared with Normal Tissue

UALCAN (http://ualcan.path.uab.edu/ accessed on 5 August 2021) is a database used to make in silico validation of target genes across cancer subtypes [36]. UALCAN provides access to publicly available OMICS data from TCGA and CPTAC. This assists researchers in identifying viable candidate genes for investigation based off of transcription and proteomic discrepancies between healthy or cancerous tissues. We used UALCAN to determine if PSMD1 and PSMD3 were significantly differentially expressed at the transcript or protein level among the cancer subtypes available in the database. The diseases analyzed in UALCAN included bladder urothelial carcinoma (BLCA), breast invasive carcinoma (BRCA), cervical squamous cell carcinoma and endocervical adenocarcinoma (CESC), cholangiocarcinoma (CHOL), colon adenocarcinoma (COAD), esophageal carcinoma (ESCA), glioblastoma multiforme (GBM), head and neck squamous cell carcinoma (HNSC), kidney chromophobe (KICH), kidney renal clear cell carcinoma (KIRC), kidney renal papillary cell carcinoma (KIRP), liver hepatocellular carcinoma (LIHC), lung adenocarcinoma (LUAD), lung squamous cell carcinoma (LUSC), pancreatic adenocarcinoma (PAAD), prostate adenocarcinoma (PRAD), pheochromocytoma and paraganglioma (PCPG), rectum adenocarcinoma (READ), sarcoma (SARC), skin cutaneous melanoma (SKCM), thyroid carcinoma (THCA), thymoma (THYM), stomach adenocarcinoma (STAD), and uterine corpus endometrial carcinoma (UCEC). The differential mRNA expression of *PSMD1* and *PSMD3* across cancer types versus normal tissue was further analyzed using Gene Expression Profiling Interactive Analysis 2 (GEPIA2) (http://gepia2.cancer-pku.cn/ accessed on 5 August 2021). GEPIA2 is another molecular analysis platform that combines data from TCGA with data from the Genotype-Tissue Expression (GTEx) project for large-scale gene expression profiling [35,37,38]. The diseases analyzed in GEPIA2 are listed in the Supplementary Materials.

### 2.2. Correlation of PSMD1 and PSMD3 mRNA Expression with Prognostic Significance across Different Cancer Types with Distinct Clinicopathological Features

In addition to gene expression analysis comparing cancerous versus normal tissues, both UALCAN and GEPIA2 provides data correlating gene expression levels with OS. We used UALCAN and GEPIA2 to determine whether high or low expression (25% cutoff) of *PSMD1* and *PSMD3* correlated with OS across TCGA cancers. Additionally, UALCAN provides mRNA expression data for different cancers comparing distinct clinicopathological features (e.g., stage). Therefore, we assessed *PSMD1* and *PSMD3* expression by tumor stage across the cancer subtypes available in the database.

### 2.3. Genomic Alterations of PSMD1 and PSMD3 across Different Cancer Types

cBioportal (https://www.cbioportal.org/ accessed on 5 August 2021) is another online platform used to explore and visualize a variety of cancer genomics data [39]. Herein, we employed the cBioportal platform to analyze the genomic abnormalities of *PSMD1* and *PSMD3* for all TCGA cancers in the database. Patients with acute myeloid leukemia (AML) were excluded for inclusion in a separate, similar analysis. The diseases analyzed in cBioportal were similar to those analyzed in UALCAN, with the addition of adrenocortical carcinoma (ACC), colon adenocarcinoma (COAD), diffuse large B-cell lymphoma (DLBCL), low grade glioma (LGG), mesothelioma (MESO), ovarian cancer (OV), tenosynovial giant cell tumor (TGCT), uterine carcinosarcoma (UCS), and uveal melanoma (UVM).

### 2.4. Statistical Analyses

Gene expression levels for *PSMD1* and *PSMD3* were calculated in UALCAN using a Student’s *t*-test, whereas GEPIA2 data were analyzed using the one-way ANOVA test, with data presented as box plots. Correlation of gene expression with OS is presented using Kaplan Meier curves. UALCAN uses a 25% cutoff for analysis of survival data. Therefore, a 25% cutoff was used for high versus low mRNA expression for both *PSMD1* and *PSMD3* in GEPIA2. For all analyses, a *p*-value of <0.05 was considered statistically significant.

## 3. Results

### 3.1. PSMD1 and PSMD3 mRNA and Protein Expression Are Upregulated in Multiple Cancer Types Compared with Normal Tissue

**Figure 1:**
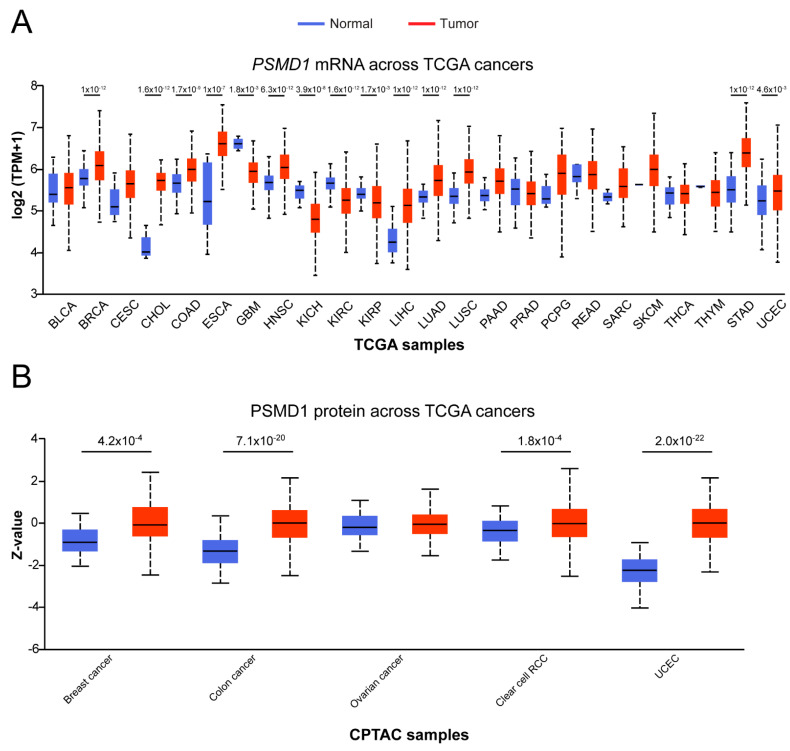
*PSMD1* expression correlates with survival in multiple human malignancies. (**A**) Box plots represent *PSMD1* mRNA expression in multiple different tumor types using TCGA RNA-seq data analyzed through UALCAN. BLCA, bladder urothelial carcinoma; BRCA, breast invasive carcinoma; CESC, cervical squamous cell carcinoma and endocervical adenocarcinoma; CHOL, cholangiocarcinoma; COAD, colon adenocarcinoma; ESCA, esophageal carcinoma; GBM, glioblastoma multiforme; HNSC, head and neck squamous cell carcinoma; KICH, kidney chromophobe; KIRC, kidney renal clear cell carcinoma; KIRP, kidney renal papillary cell carcinoma; LIHC, liver hepatocellular carcinoma; LUAD, lung adenocarcinoma; LUSC, lung squamous cell carcinoma; PAAD, pancreatic adenocarcinoma; PRAD, prostate adenocarcinoma; PCPG, pheochromocytoma and paraganglioma; READ, rectum adenocarcinoma; SARC, sarcoma; SKCM, skin cutaneous melanoma; THCA, thyroid carcinoma; THYM, thymoma; STAD, stomach adenocarcinoma; UCEC, uterine corpus endometrial carcinoma. (**B**) PSMD1 protein expression in different tumor types compared with normal tissue using CPTAC data analyzed through UALCAN. RCC, renal cell carcinoma; UCEC, uterine corpus endometrial carcinoma.

We recently demonstrated that knockdown of PSMD1 and PSMD3 resulted in increased apoptosis of CML cells but not normal cord blood controls, implicating they may be good targets for therapy [31]. Thus, we hypothesized that their relevance stretches beyond CML to other types of cancers and that they may be targets for anti-cancer therapeutics. To address this hypothesis, we analyzed PSMD1 and PSMD3 mRNA and protein expression using TCGA and CPTAC data available at UALCAN [36]. At the mRNA level, *PSMD1* was found to be significantly differentially expressed in 14/24 (58%) TCGA cancers compared with normal controls (Figure 1A).

Cancers demonstrating higher *PSMD1* mRNA expression compared with controls included BRCA, which is consistent with previous reports [20,22]. Additional cancers included CHOL, COAD, ESCA, HNSC, LIHC, LUAD, LUSC, STAD, and UCEC (Figure 1A). In contrast, patients with GBM, KICH, KIRC, and KIRP demonstrated reduced *PSMD1* mRNA expression compared with control tissue (Figure 1A). At the protein level, PSMD1 was found to be upregulated in 4/5 (80%) CPTAC cancers [35], including breast cancer, colon cancer, clear cell renal cell carcinoma (RCC), and UCEC (Figure 1B).

**Figure 2:**
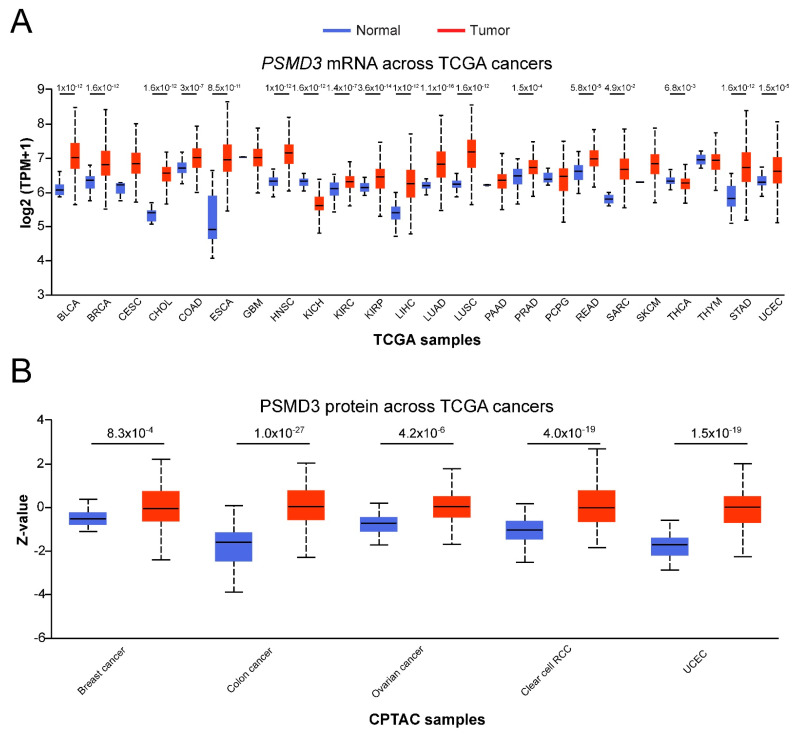
*PSMD3* expression correlates with survival in multiple human malignancies. (**A**) Box plots represent *PSMD3* mRNA expression in multiple different tumor types using TCGA RNA-seq data analyzed through UALCAN. BLCA, bladder urothelial carcinoma; BRCA, breast invasive carcinoma; CESC, cervical squamous cell carcinoma and endocervical adenocarcinoma; CHOL, cholangiocarcinoma; COAD, colon adenocarcinoma; ESCA, esophageal carcinoma; GBM, glioblastoma multiforme; HNSC, head and neck squamous cell carcinoma; KICH, kidney chromophobe; KIRC, kidney renal clear cell carcinoma; KIRP, kidney renal papillary cell carcinoma; LIHC, liver hepatocellular carcinoma; LUAD, lung adenocarcinoma; LUSC, lung squamous cell carcinoma; PAAD, pancreatic adenocarcinoma; PRAD, prostate adenocarcinoma; PCPG, pheochromocytoma and paraganglioma; READ, rectum adenocarcinoma; SARC, sarcoma; SKCM, skin cutaneous melanoma; THCA, thyroid carcinoma; THYM, thymoma; STAD, stomach adenocarcinoma; UCEC, uterine corpus endometrial carcinoma. (**B**) PSMD3 protein expression in different tumor types compared with normal tissue, as determined by CPTAC data analyzed through UALCAN [36]. RCC, renal cell carcinoma; UCEC, uterine corpus endometrial carcinoma.

*PSMD3*, on the other hand, was found differentially expressed at the mRNA level in 18/24 (75%) TCGA cancers (Figure 2A). Cancers with increased *PSMD3* expression included BLCA, BRCA, CHOL, COAD, ESCA, HNSC, KIRC, KIRP, LIHC, LUAD, LUSC, PRAD, READ, SARC, STAD, and UCEC (Figure 2A). Patients with KICH or THCA, in contrast, demonstrated reduced levels of *PSMD3* in malignant versus normal tissue (Figure 2A). At the protein level, PSMD3 was found to be upregulated in 5/5 (100%) CPTAC cancers, including breast cancer, colon cancer, ovarian cancer, clear cell RCC, and UCEC (Figure 2B).

Altogether, these data confirm that PSMD1 and PSMD3 mRNA and protein are differentially expressed in multiple types of human cancers, similar to our recent findings in CML disease progression and TKI resistance [31].

### 3.2. Genomic Alterations of PSMD1 and PSMD3 in Cancer

**Figure 3:**
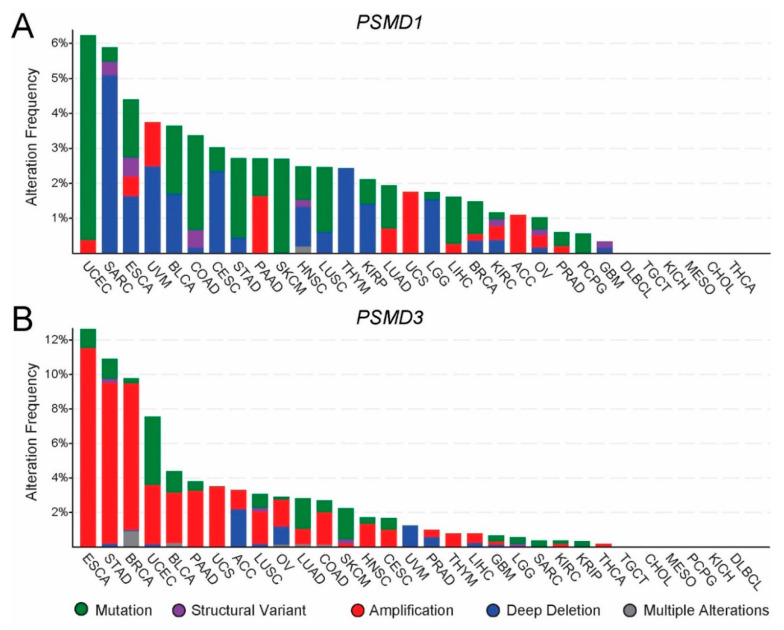
Genomic alterations in the genes encoding PSMD1 and PSMD3 in multiple human cancers. Using data from cBioPortal, we analyzed genomic alterations associated with the *PSMD1* (**A**) and *PSMD3* (**B**) genes in multiple TCGA cancers. The *PSMD1* gene was primarily associated with mutations or deep deletions, whereas the *PSMD3* gene showed mostly amplifications. ACC, adrenocortical carcinoma; BLCA, bladder urothelial carcinoma; BRCA, breast invasive carcinoma; CESC, cervical squamous cell carcinoma and endocervical adenocarcinoma; CHOL, cholangiocarcinoma; COAD, colon adenocarcinoma; DLBCL, lymphoid neoplasm diffuse large B-cell lymphoma; ESCA, esophageal carcinoma; GBM, glioblastoma multiforme; HNSC, head and neck squamous cell carcinoma; KICH, kidney chromophobe; KIRC, kidney renal clear cell carcinoma; KIRP, kidney renal papillary cell carcinoma; LGG, Brain lower grade glioma; LIHC, liver hepatocellular carcinoma; LUAD, lung adenocarcinoma; LUSC, lung squamous cell carcinoma; MESO, mesothelioma; OV, ovarian serous cystadenocarcinoma; PAAD, pancreatic adenocarcinoma; PRAD, prostate adenocarcinoma; PCPG, pheochromocytoma and paraganglioma; READ, rectum adenocarcinoma; SARC, sarcoma; SKCM, skin cutaneous melanoma; TGCT, testicular germ cell tumors; THCA, thyroid carcinoma; THYM, thymoma; STAD, stomach adenocarcinoma; UCEC, uterine corpus endometrial carcinoma; UCS, uterine carcinosarcoma; UVM, uveal melanoma.

We next analyzed genomic alterations of the genes encoding PSMD1 and PSMD3 using cBioportal [39]. Overall, *PSMD1* DNA is prone to mutations or deep deletions, whereas *PSMD3* DNA is primarily prone to amplifications (Figure 3A,B). PSMD1 showed mutations in diseases such as UCEC, BLCA, COAD, STAD, SKCM, LUAD, LIHC, and BRCA, and deep deletions in diseases like SARC, ESCA, UVM, BLCA, CESC, HNSC, THYM, KIRC, and LGG (Figure 3A). *PSMD3*, on the other hand, demonstrated amplifications in ESCA, STAD, BRCA, UCEC, BLCA, PAAD, UCS, LUSC, OV, COAD, HNSC, and CESC (Figure 3B).

Altogether, mutations, amplification, or deep deletions in the genes encoding PSMD1 and PSMD3 could explain their differential expression in different types of cancers.

### 3.3. High Expression of PSMD1 or PSMD3 mRNA Correlates with OS in Numerous Different Cancers

**Figure 4:**
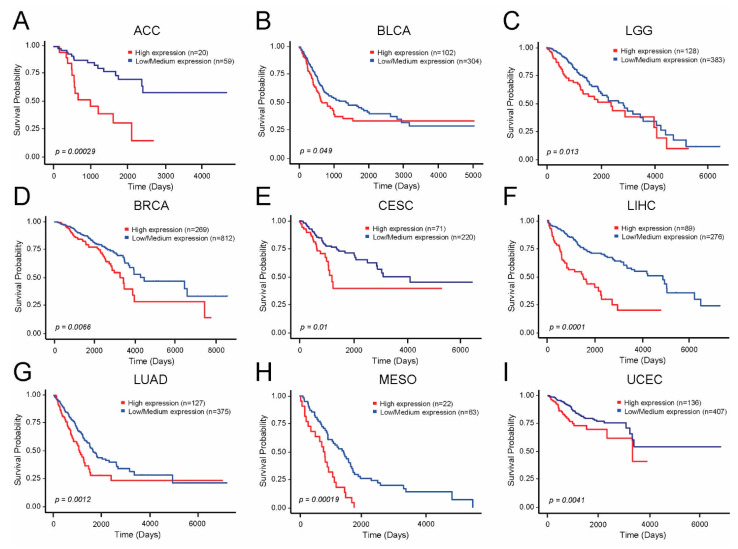
*PSMD1* expression correlates with worse OS in multiple human malignancies. TCGA survival data from UALCAN demonstrates that higher levels of *PSMD1* mRNA expression correlated with worse outcomes as demonstrated in the Kaplan-Meier curves for (**A**) ACC, adrenocortical carcinoma; (**B**) BLCA, bladder urothelial carcinoma; (**C**) LGG, Brain lower grade glioma; (**D**) BRCA, breast invasive carcinoma; (**E**) CESC, cervical squamous cell carcinoma and endocervical adenocarcinoma; (**F**) LIHC, liver hepatocellular carcinoma; (**G**) LUAD, lung adeno carcinoma; (**H**) MESO, mesothelioma; (**I**) UCEI, uterine corpus endometrial carcinoma.

The observation that *PSMD1* and *PSMD3* are upregulated in multiple different cancer types suggested that they may also play a role in outcomes. We next sought to determine whether expression of *PSMD1* or *PSMD3* correlates with OS in various cancers. Using data available at UALCAN, higher expression of *PSMD1* mRNA correlated with reduced OS in multiple cancer types, including ACC, BLCA, LGG, BRCA, CESC, LIHC, LUAD, MESO, and UCEC (Figure 4A–I).

**Figure 5:**
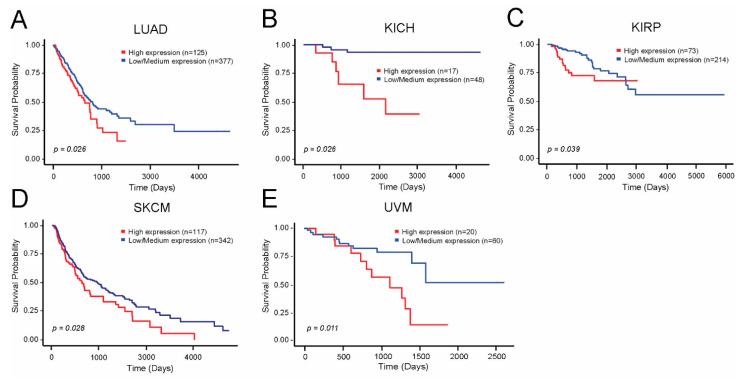
*PSMD3* expression correlates with worse OS in multiple human malignancies. TCGA survival data from UALCAN demonstrates that higher levels of *PSMD3* mRNA expression correlated with worse outcomes as demonstrated in the Kaplan-Meier curves for (**A**) LUAD, lung adeno carcinoma; (**B**) KICH, kidney chromophobe; (**C**) KIRP, kidney renal papillary cell carcinoma; (**D**) SKCM, skin cutaneous melanoma; (**E**) UVM, uveal melanoma.

Similarly, higher expression of *PSMD3* mRNA correlated with reduced OS in patients with LUAD, KICH, KIRP, SKCM, and UVM (Figure 5A–E). High levels of *PSMD3* mRNA expression also correlated with worse OS in patients with AML, which will be presented in a similar, separate analysis (data not shown).

Altogether, these data confirm that higher expression of *PSMD1* and *PSMD3* correlates with OS in a number of different human malignancies.

To corroborate these findings, we also correlated expression of these genes with OS using GEPIA2 [38], which combines TCGA [33] and GTEx [35,37] data for larger patient cohorts. On this platform, *PSMD1* mRNA correlated with worse OS in patients with ACC, LGG, LUAD, MESO, and UVM (Figure S1A–E). In contrast, patients with DLBCL or STAD who had higher levels of *PSMD1* mRNA expression demonstrated a better OS (Figure S1F,G). For the cancers that demonstrated a significant difference in OS using GEPIA2, we also analyzed expression levels in malignant versus normal tissue. Using GEPIA2, only patients with STAD and DLBCL demonstrated higher levels of *PSMD1* mRNA in malignant versus control tissue (Figure S1H,I). This is despite the fact that high *PSMD1* correlated with better OS in either disease (Figure S1F,G). High levels of *PSMD3* mRNA expression, in contrast, correlated with a worse OS in MESO and a better OS in thymoma (THYM) (Figure S2A,B). Similar to DLBCL and STAD, patients with THYM demonstrated higher levels of *PSMD3* mRNA expression compared with normal controls, despite lower levels correlating with worse outcomes (Figure S2C).

Altogether, these data indicate that high expression levels of *PSMD1* and *PSMD3* mRNA correlate with worse outcomes in multiple different cancers, with the exception of patients with DLBCL, STAD, and THYM.

### 3.4. Correlation of PSMD1 and PSMD3 mRNA Expression with Distinct Clinicopathological Features in Certain Types of Cancers

**Figure 6:**
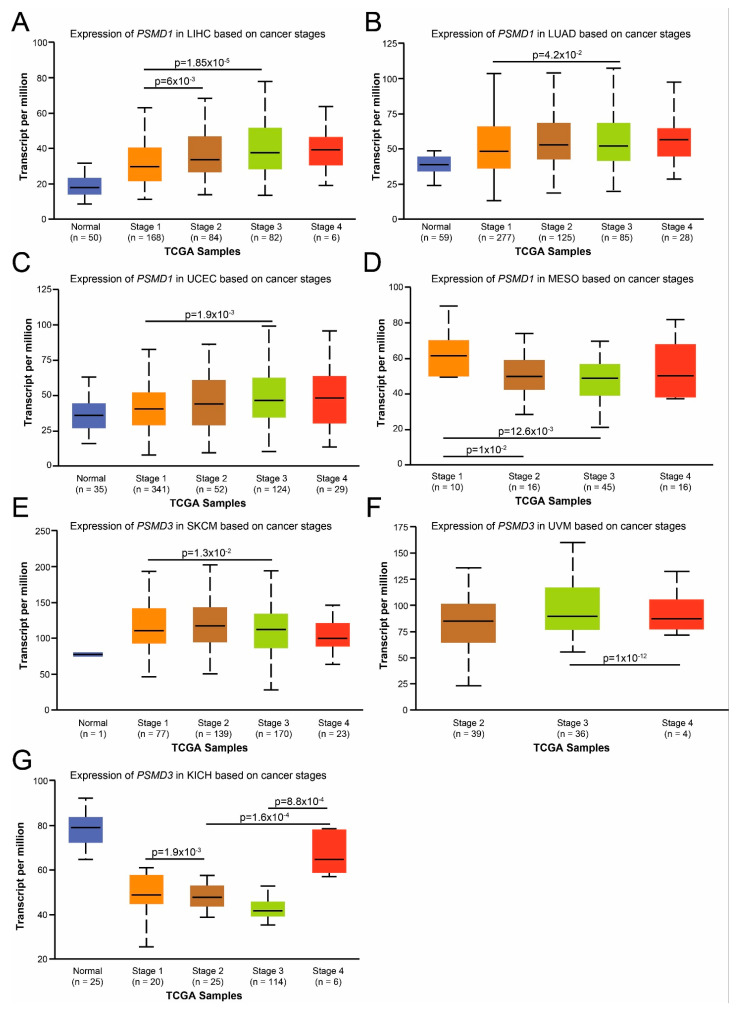
Correlation of *PSMD1* and *PSMD3* mRNA expression with distinct clinicopathological features in certain types of cancers. We used UALCAN to associate *PSMD1* and *PSMD3* mRNA expression with clinicopathological characteristics. *PSMD1* mRNA expression was significantly increased in patients with LIHC (**A**), LUAD (**B**), and UCEC (**C**) comparing disease stages 1, 2, or 3. *PSMD1* mRNA expression was reduced in patients with MESO when comparing stages 1, 2, and 3 (**D**). In patients with SKCM, *PSMD3* mRNA levels decreased when comparing stage 1 with stage 3 (**E**). In patients with UVM, *PSMD3* mRNA levels also decreased when comparing stage 3 with stage 4 (**F**). In patients with KICH, while *PSMD3* mRNA levels decreased comparing normal specimens with stages 1, 2, or 3, it was upregulated in patients who had progressed to stage 4 (**G**).

For the cancers demonstrating significant differences in OS, we next associated *PSMD1* and *PSMD3* mRNA expression with clinicopathological characteristics using UALCAN. *PSMD1* mRNA expression was significantly increased in patients with LIHC, LUAD, and UCEC comparing disease stages 1, 2, or 3 (Figure 6A–C). In contrast, *PSMD1* mRNA expression was reduced in patients with MESO in stages 1, 2, and 3 (Figure 6D). *PSMD3* expression was somewhat different. In patients with SKCM, *PSMD3* mRNA levels decreased when comparing stage 1 with stage 3 (Figure 6E). In patients with UVM, *PSMD3* mRNA levels also decreased when comparing stage 3 with stage 4 (Figure 6F). Interestingly, in patients with KICH, while *PSMD3* mRNA levels decreased comparing normal specimens with stages 1, 2, and 3, it was markedly upregulated in KICH patients who had progressed to stage 4 (Figure 6G). For all other cancers demonstrating significant differences in OS, *PSMD1* or *PSMD3* mRNA expression had no correlation with disease stage (data not shown).

Altogether, these findings suggest that *PSMD1* and *PSMD3* mRNA expression could serve as novel prognostic biomarkers for cancers affecting multiple different tissue types, especially for patients with KICH who are progressing from stage 3 to stage 4 of the disease [31].

## 4. Discussion

The development of public databases such as TCGA, UALCAN, cBioPortal, and GEPIA2 have greatly improved the ability of biomedical scientists to interrogate the role of genes and proteins in cancer initiation, progression, and relapse [40]. In the present study, we used publically available databases to assess the effects of altered PSMD1 and PSMD3 mRNA and protein expression on disease progression and OS for multiple different cancers. Ultimately, our analysis demonstrated that PSMD1 and PSMD3 mRNA and protein were markedly upregulated in numerous different cancers, which correlated with a worse OS, indicating they may serve as novel prognostic biomarkers in cancer diagnosis and therapy response.

We propose that PSMD1 and PSMD3, both subunits of the 19S regulatory complex of the 26S proteasome, are proteins worthy of further investigation for cancer prognosis and therapy. From our previous work with these proteins in CML [31], and what has been published previously in HER2+ breast cancer [22,23], we decided to uncover their relevance as biomarkers or as therapeutic targets in other types of cancers. Our analysis using TCGA and CPTAC data in UALCAN showed that there are significant differences of expression at the mRNA and protein level in a variety of cancers. When TCGA data is correlated with OS for *PSMD1* and *PSMD3,* there were notable examples of worse OS when the genes were more highly expressed. Interestingly, this is contrary to a previous report by Tsvetkov et al., who demonstrated that reduced expression of several different 19S proteasome subunits, including *PSMD1, PSMD5, PSMD6,* or *PSMD10*, correlated with higher relapse rates and a worse OS in patients with multiple myeloma [41]. In our analysis, however, some of the cancer types showed a similar phenotype, demonstrating decreased OS with decreased *PSMD1* or *PSMD3* expression. For example, our data showed that patients with DLBCL or STAD who had higher levels of *PSMD1* expression showed increased OS (Figure S1F,G). Similarly, patients with THYM who had higher levels of *PSMD3* expression also showed a better OS (Figure S2). Despite reduced expression correlating with worse outcomes, patients with DLBCL or STAD still showed significantly higher levels of *PSMD1* mRNA compared with normal controls (Figure S1F,G), and patients with THYM showed higher levels of *PSMD3* compared with controls (Figure S2C). Thus, there appears to be tissue-specific differences on the phenotype resulting from altered expression of PSMD1 or PSMD3 in different cancer types.

UALCAN’s TCGA data and the genomic alteration data from cBioportal are worth examining in concert, in order to determine the mechanism by which these genes are differentially expressed in the observed cancers. Indeed, mutations at the promoter region of *PSMD1* and *PSMD3* could be an explanatory mechanism for their altered mRNA abundance. Alternatively, during oncogenesis as cancer cells rapidly divide, the increase in gene expression could be due to gene amplifications, where more copies of *PSMD1* and *PSMD3* are erroneously introduced during replication. Interestingly, data from cBioportal demonstrated that the gene encoding PSMD1 is primarily prone to mutations and deep deletions, but also occasionally with gene amplifications (Figure 3A). In patients with ACC, for example, who demonstrated a reduced OS with high levels of *PSMD1* expression (Figure 4A), showed exclusively amplifications as the main genomic alteration (Figure 3A).

In contrast, patients with GBM, which demonstrated reduced *PSMD1* expression compared with normal controls (Figure 1A), were associated with structural variants and deep deletions (Figure 3A), possibly explaining the reduced expression observed in this particular type of cancer.

The gene encoding PSMD3, in contrast, is primarily associated with amplifications (Figure 3B). For example, patients with ESCA or STAD demonstrate increased *PSMD3* mRNA expression compared with normal controls (Figure 2A), which correlated with a high degree of gene amplifications (Figure 3B). On the other hand, patients with KICH demonstrated reduced *PSMD3* mRNA expression compared with controls (Figure 2A). However, no genomic alterations have been identified to date (Figure 3B). In this particular case, altered expression of PSMD3 could be due to post-transcriptional, translational, or post-translational regulation. We previously speculated that the difference in sensitivity between CML and normal progenitors to knockdown of PSMD1 or PSMD3 may be due to differences in post-translational modifications, which could alter peptide targets in malignant versus normal tissues [31]. Indeed, Hemmis et al. recently reported that PSMD1 can be phosphorylated at tyrosine 950, and that this modulates interactions between ubiquitin receptors [42].

The post-translational modifications of PSMD1 or PSMD3 could provide a starting point for targeting these proteins in cancer therapy. In addition to phosphorylation events [42], PSMD1 was reported to undergo SUMOylation, the addition of small ubiquitin-related modifiers [43]. The SUMOylation event occurs at a critical lysine residue located immediately adjacent to the ADRM1-binding domain, an ubiquitin receptor [44], thereby regulating associations between the two proteins [43]. Importantly, ADRM1/RPN13 has already been implicated as a potential target for cancer therapy [45,46,47,48,49,50,51,52,53,54]. Less is known about PSMD3 post-translational modifications. However, in yeast, PSMD3 (RPN3) was shown to interact with RPN11, and *PSMD3* mutants resulted in a proteasome assembly defect [55]. Therefore, this interaction may potentially be subject to pharmacologic intervention. The post-translational modifications of PSMD1 or PSMD3, the proteins they interact with, and the enzymes that regulate them could provide a mechanism for pharmacologic inhibition in cancer therapy.

Future studies will determine the mechanism by which PSMD1 or PSMD3 are upregulated in CML and other types of cancers, and will interrogate their potential as novel therapeutic targets. Future studies will also identify the mechanism by which they act as oncogenes in certain types of malignancies. Our recent data in CML suggest that PSMD1 and PSMD3 act as oncogenes by stabilizing the nuclear factor-kappa B (NF-κB) transcription factor from degradation [31]. Similarly, these proteasome subunits were shown to regulate p53 and HER2 degradation in breast cancer [22,23]. We speculate that other signaling pathways are regulated by these proteins in different types of cancers, and that those pathways will have relevance to cancer initiation, progression, and relapse, depending on the cell and tissue of origin. PSMD1, PSMD3, and the signaling pathways regulated by them, could be novel molecular targets for improved cancer therapy. Our observation that knockdown of these proteins reduced survival of CML but not normal progenitors suggests they may be good molecular targets with less toxicity compared with traditional proteasome inhibitors [31]. In order to target PSMD1 or PSMD3 directly, we must first have a better understanding of their post-translational modifications, the proteins that interact with them (e.g., deubiquitylases), and their differences between normal versus malignant cells.

Altogether, our data implicate PSMD1 and PSMD3 as novel potential prognostic biomarkers and therapeutic targets that are worthy of future investigation.

## Supplementary Materials

The following are available online at https://www.mdpi.com/article/10.3390/cells10092390/s1, Figure S1: *PSMD1* expression correlates with worse OS in multiple human malignancies, Figure S2: *PSMD3* expression correlates with worse OS in multiple human malignancies.
