# Supplementary material for: 26S Proteasome Non-ATPase Regulatory Subunits 1 (PSMD1) and 3 (PSMD3) as Putative Targets for Cancer Prognosis and Therapy
Source: Cells. 2021 Sep 11;10(9):2390. doi: 10.3390/cells10092390 (PMC8472613; doi:10.3390/cells10092390)
Supplement: Supplementary file 1 [file cells-10-02390-s001.zip › cells-1351066-supplementary.pdf]

26S proteasome non-ATPase regulatory subunits 1 (PSMD1) and 3 (PSMD3) as putative targets for cancer prognosis and therapy

Andres J. Rubio[1], Alfonso E. Bencomo-Alvarez[2], James E. Young[3], Vanessa V. Velazquez[1], Joshua J. Lara[1], Mayra A. Gonzalez[2], Anna M. Eiring[1-3*]

[1]Paul L. Foster School of Medicine, Texas Tech University Health Sciences Center at El Paso, El Paso, TX, USA; [2]Center of Emphasis in Cancer, Department of Molecular and Translational Medicine, Paul L. Foster School of Medicine, Texas Tech University Health Sciences Center at El Paso, El Paso, TX, USA; [3]Graduate School of Biomedical Sciences, Texas Tech University Health Sciences Center at El Paso, El Paso, TX, USA.

[*]Corresponding Author: Anna M. Eiring, PhD; 5001 El Paso Drive, MSC 32002, MSB1 Room 2112, El Paso, TX 79905, USA; Ph: (915) 215-4812; E-Mail: anna.eiring@ttuhsc.edu

Abstract: Ever since the ubiquitin proteasome system was characterized, efforts have been made to manipulate its function to abrogate the progression of cancer. As a result, the anticancer drugs bortezomib, carfilzomib, and ixazomib targeting the 26S proteasome were developed to treat multiple myeloma, mantle cell lymphoma, and diffuse large B-cell lymphoma, among others. Despite success, adverse side effects and drug resistance are prominent, raising the need for alternative therapeutic options. We recently demonstrated that knockdown of the 19S regulatory components, proteasome 26S subunit, non-ATPases 1 (*PSMD1*) and 3 (*PSMD3*), resulted in increased apoptosis of chronic myeloid leukemia (CML) cells, but had no effect on normal controls, suggesting they may be good targets for therapy. Therefore, we hypothesized that PSMD1 and PSMD3 are potential targets for anticancer therapeutics and that their relevance stretches beyond CML to other types of cancers. In the present study, we analyzed PSMD1 and PSMD3 mRNA and protein expression in cancerous tissue versus normal controls using data from The Cancer Genome Atlas (TCGA) and the Clinical Proteomic Tumor Analysis Consortium (CPTAC), comparing expression with overall survival. Altogether, our data suggest that PSMD1 and PSMD3 may be novel putative targets for cancer therapy that are worthy of future investigation.

Keywords: Oncogenes, drug targets, proteasome inhibition

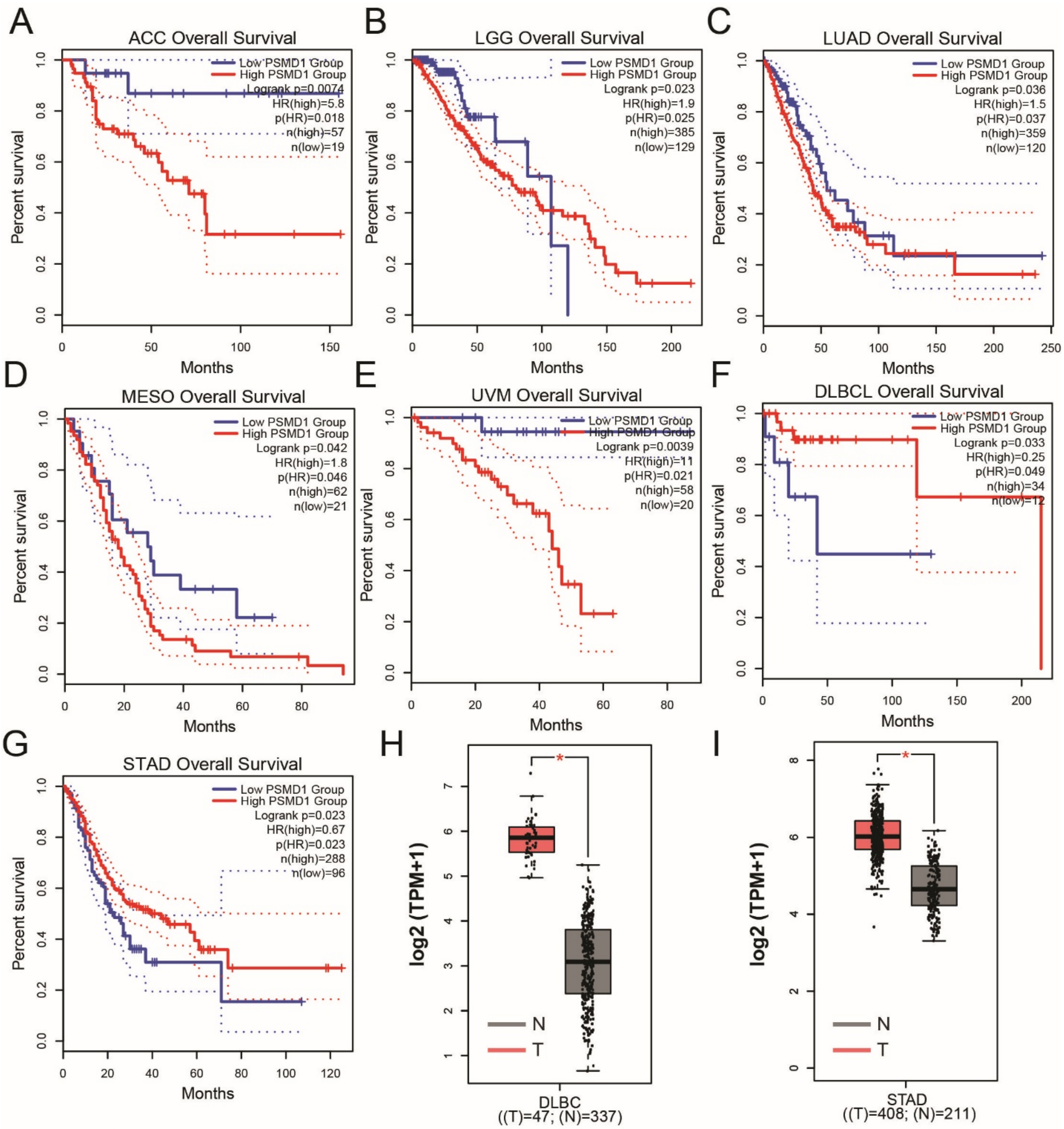

Figure S1. *PSMD1* expression correlates with worse overall survival (OS) in multiple human malignancies. Survival data from GEPIA2 demonstrates that higher levels of *PSMD1* mRNA expression correlated with worse outcomes as demonstrated in the Kaplan-Meier curves for (**A**) ACC, adrenocortical carcinoma; (**B**) LGG, lower grade glioma; (**C**) LUAD, lung adeno carcinoma; (**D**) MESO, mesothelioma; and (**E**) UVM, uveal melanoma. In contrast, lower levels of *PSMD1* mRNA expression correlated with a worse OS in (**F**) DLBCL, diffuse large B-cell lymphoma; and (**G**) STAD, stomach adenocarcinoma. Despite lower levels of expression correlating with worse overall survival, patients with DLBCL (**H**) and STAD (**I**) demonstrated higher levels of *PSMD1* expression compared with normal controls.

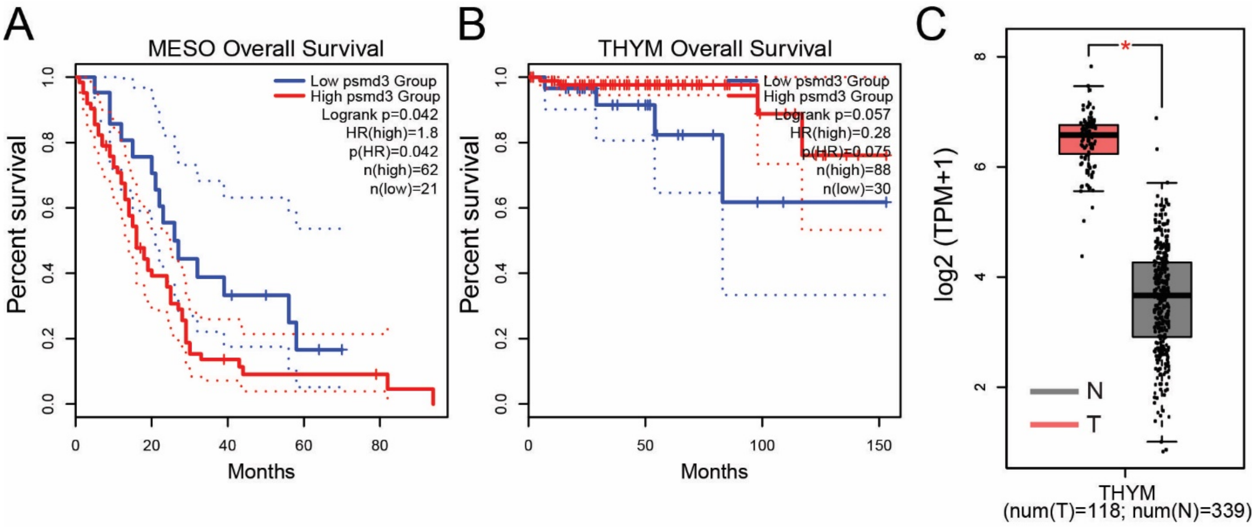

Figure S2. *PSMD3* expression correlates with worse OS in multiple human malignancies. Survival data from GEPIA2 demonstrated that higher levels of *PSMD3* mRNA expression correlated with worse outcomes in the Kaplan-Meier curves for (**A**) MESO, mesothelioma, but better outcomes in (**B**) THYM, thymoma. Despite lower levels of expression correlating with worse overall survival, patients with THYM (**C**) demonstrated higher levels of *PSMD3* expression compared with normal controls.
